# Nationwide Seropositivity of Hepatitis A in Republic of Korea from 2005 to 2014, before and after the Outbreak Peak in 2009

**DOI:** 10.1371/journal.pone.0170432

**Published:** 2017-01-18

**Authors:** Kyung-Ah Kim, Anna Lee, Moran Ki, Sook-Hyang Jeong

**Affiliations:** 1 Departments of Internal Medicine, Inje University Ilsan Paik Hospital, Goyang, Republic of Korea; 2 Department of Laboratory Medicine, Seoul Clinical Laboratories, Yongin, Republic of Korea; 3 Department of Cancer Control and Policy, Graduate School of Cancer Science and Policy, National Cancer Center, Goyang, Republic of Korea; 4 Departments of Internal Medicine, Seoul National University Bundang Hospital, College of Medicine, Seoul National University, Seongnam, Republic of Korea; Centers for Disease Control and Prevention, UNITED STATES

## Abstract

**Background/Aims:**

The epidemiologic shift of hepatitis A virus (HAV) infection in the South Korean population resulted in a peak outbreak of hepatitis in 2009. The aim of this study was to clarify the seropositivity of anti-HAV antibody (anti-HAV) and its demographic characteristics before and after the peak outbreak from 2005 to 2014.

**Methods:**

This retrospective study analyzed the anti-HAV data of all individuals from 1,795 medical institutions referred to a major central laboratory from January 2005 through December 2014, as a sentineal tool for monitoring annual variation of anti-HAV positivity. The prevalence of anti-HAV was adjusted for age and area with the standard population based on the 2010 Census data.

**Results:**

A total of 424,245 individuals were included in this study. The overall age-adjusted anti-HAV prevalence decreased from 65.6% in 2005 to 62.2% in 2014. During the 10-year period, the seroprevalence continuously decreased in persons aged 30 to 39 years (69.6% to 32.4%) and those aged 40 to 49 years (97.9% to 79.3%) due to the cohort effect. In contrast, it increased in persons aged 10 to 19 years (15.4% to 35.2%), while it was the lowest (8.7%) in 2010 before rebounding to 20.2% in 2014 in persons aged 20 to 29 years due to a vaccination effect.

**Conclusion:**

Although the HAV vaccination rate increased, the anti-HAV seropositivity in South Korea decreased from 65.6% to 62.2% in this study population. In particular, the immunity of young adults was still low, and an outbreak of HAV is possible in the near future. Therefore, continuous monitoring and optimal preventive measures to prevent future outbreaks should be considered.

## Introduction

Hepatitis A virus (HAV) is a positive-strand RNA virus causing fecal-orally transmitted acute viral hepatitis.[1] Though an effective hepatitis A vaccine was introduced in the mid-1990s, according to the World Health Organization global disease burden epidemiology reference group, HAV resulted in approximately 1.4 million cases worldwide annually and 27,731 deaths in 2010, of which South-East Asian regions including South Korea showed the highest disease burden of HAV.[2]

During the past decade, significant changes in HAV epidemiology have been noticed, closely related to the hygiene, sanitary conditions and socio-economic levels of many countries. This epidemiological shift showing lower HAV seroprevalence among children and young adults resulted in an increased risk for HAV outbreak and increased morbidity.[3]

As a result of the HAV epidemiological shift, South Korea experienced a large nationwide outbreak of HAV from 2007–2011[4], during which the peak incidence was recorded in 2009 [15,231 cases from the sentinel surveillance system data of the Korean Centers for Disease Control and Prevention (KCDC)][5]. Hepatitis A was reported mostly frequently in individuals in their thirties (50.0%) followed by twenties (37.2%) and forties (9.9%) in 2009. After 2009, hepatitis A incidence decreased drastically, and only 867 cases were reported to the KCDC in 2013. Following this epidemic, the KCDC added HAV to the national immunization program for children since May 2015. However, the dynamic changes of seroprevalence before and after the nationwide outbreak needs to be monitored and evaluated.

It is important to know the age-specific seroprevalence of HAV collected every 5 or 10 years to assess population immunity and susceptibility. The aim of this study was to clarify the nationwide seroprevalence of anti-HAV in Korea from 2005 to 2014 by age and region using the serologic results of HAV from a major central laboratory as a sentinel tool for monitoring annual variations of anti-HAV positivity.

## Methods

### Materials

We analyzed anonymous data on the serological results for HAV that were referred to Seoul Clinical Laboratories (SCL) from 1,795 nationwide medical institutions nationwide in Korea between 2005 and 2014, as a sentinel tool for monitoring annual variation of anti-HAV positivity. Both total and immunoglobulin G (IgG) anti-HAV positive sera were considered to be positive for anti-HAV in this study. SCL is a major central laboratory responsible for 22.4% of nationwide HAV serologic tests, based on data from the Health Insurance Review and Assessment Service (HIRA) of Korea between 2010 and 2014. Because the data were analyzed anonymously, informed consents were not obtained based on SLC regulation. This study was conducted according to the principles expressed in the Declaration of Helsinki, and approved by the Institutional Review Board of SCL (SCL-IRB-201503).

From January 2005 to December 2009, the results of total anti-HAV tests from 25,140 subjects (1,140 in 2005, 1,642 in 2006, 2,050 in 2007, 6,207 in 2008 and 14,101 in 2009, respectively) were analyzed based on 2005 national census data, and through previously published methods identical to that of this study [6]. Due to heightened awareness about HAV during the nationwide outbreak of hepatitis A, far more doctors and patients requested anti-HAV tests, and therefore, the sample numbers rapidly increased during this study period.

From January 2010 to December 2014, a total of 654,415 individuals’ sera were referred to SCL for anti-HAV tests. Among these, the following cases were excluded: 2,800 with positive IgM anti-HAV; 44,705 with only IgM anti-HAV results; and 207,805 with total or IgG anti-HAV results but no demographic information. Therefore, 399,105 requested individuals with demographic information were included in this study (60,846 in 2010, 83,586 in 2011, 76,906 in 2012, 84,102 in 2013 and 93,665 in 2014, respectively). The crude anti-HAV positivity of the enrolled 399,105 individuals (52.0%) was not different from the 207,805 individuals without demographic information (52.1%), which suggested that exclusion of the individuals without demographic information may have had little influence on the overall results of this study.

### Anti-HAV test

Total anti-HAV tests were performed with electro-chemiluminescence immunoassay using the Modular Analytics E170 system (Roche Diagnostics, Mannheim, Germany). IgG and IgM anti-HAV tests were performed with chemiluminescent microparticle immunoassay using the ARCHITECT *i*2000 system (Abbott Laboratories, Wiesbaden, Germany).

### Statistical analysis

Anti-HAV prevalence calculated by both total and IgG anti-HAV positivity was analyzed by age and area. Subjects were grouped into ages by ten-year intervals and were also grouped into 6 administrative areas including Seoul, Gyeonggi/Incheon, Gangwon, Chungcheong, Gyeongsang, and Jeolla/Jeju. Nationwide seroprevalence of HAV was adjusted by age, area, and sex (only for the latter 5 years because the limited sample size during the first 5 years did not permit gender-adjusted analysis) with the standard population based on the 2010 national census data of South Korea. Age-specific seroprevalence was adjusted according to area and gender, and area-specific seroprevalence was adjusted with age and gender in the same way. Statistical analysis was conducted with SPSS for Windows (version 21.0, SPSS Inc., Chicago, IL, USA) and the positive rates by area and year were compared via the Pearson chi-square test. A *p*-value of less than 0.05 was considered to indicate a statistically significant difference.

## Results

### Characteristics of the study population

The total subject demographics from 2005 to 2014 are summarized in Table 1. For 25,140 subjects enrolled during 2005–2009, the mean age was 37.4 years and the male-to-female ratio was 1:0.99 as previously reported.[6] For 399,105 subjects enrolled during 2010–2014, the mean age was 37.8 years and the male-to-female ratio was 1:1.42. The male-to-female ratio was reversed in the latter half of the period, probably due to increased prenatal screening of IgG anti-HAV for women of child-bearing age after the outbreak peaked in 2009.

**Table 1:** Characteristics of study population.

Variable	Year									
	2005	2006	2007	2008	2009	2010	2011	2012	2013	2014
Number	1,140	1,642	2,050	6,207	14,101	60,846	83,586	76,906	84,102	93,665
Age	31.0±20.4	34.1±18.4	35.7±17.7	37.6±17.4	38.4±14.9	37.5±12.8	37.5±12.6	37.7±12.9	38.1±12.9	37.8±12.4
Male-to-female ratio	1:0.76	1:0.82	1:0.74	1:1.00	1:0.93	1:1.12	1:1.35	1:1.64	1:1.49	1:1.49
Age group										
0~9	231	158	151	401	294	787	613	1,130	1,108	854
10~19	141	224	245	445	1,049	3,313	3,601	3,068	2,707	3,007
20~29	171	314	347	1,217	2,500	11,996	16,299	14,008	15,011	17,529
30~39	225	341	470	1,540	4,191	20,528	30,902	29,321	32,231	37,263
40~49	156	265	393	1,203	2,991	13,162	17,495	14,918	17,191	18,252
50~59	104	180	255	775	1,819	7,854	10,041	10,118	10,757	11,607
60+	112	16	189	716	1,257	3,206	4,635	4,343	5,097	5,153

### The changing pattern of anti-HAV seroprevalence from 2005 to 2014

The age-adjusted anti-HAV prevalence overall showed a gradual decreasing tendency from 2005 to 2014 as summarized in Table 2 (65.6% in 2005, 63.8% in 2009, and 62.2% in 2014, respectively). Considering the experience of nationwide outbreak and the active vaccination campaign during the outbreak, this decrease of overall anti-HAV prevalence was unexpected. It seemed to result primarily from the cohort effect as persons who were born between mid- 1970s and mid-1990s did not acquire natural immunity via subclinical HAV infection during their early childhood.

**Table 2:** Area and sex-adjusted seroprevalence of anti-HAV from 2005 to 2014*.

Age (year)	2005	2006	2007	2008	2009	2010	2011	2012	2013	2014
0–9	33.4	52.7	42.4	50.7	69.8	65.8	53.9	71.0	65.4	67.7
10~19	15.4	19.0	20.0	25.5	23.2	19.0	26.4	33.8	35.4	35.2
20~29	22.5	29.2	19.1	17.2	11.9	8.7	13.3	16.2	16.3	20.2
30~39	69.6	67.6	63.8	58.6	48.4	40.7	37.3	34.3	32.1	32.4
40~49	97.9	96.7	94.7	91.4	89.1	87.9	86.1	84.1	80.8	79.3
50~59	98.7**	98.2**	98.0**	99.0**	98.8**	98.7	98.7	98.4	98.0	98.1
60~						99.2	98.1	99.3	99.5	99.6
Overall	65.6	68.2	64.9	65.1	63.8	61.0	60.5	62.9	61.6	62.2

HAV, hepatitis A virus.

* Seroprevalence of anti-HAV was adjusted by area from 2005 to 2009 and by area and gender from 2010 to 2014

**Persons in their fifties and over sixty were grouped together from 2005 to 2009 because of the small number and similar seroprevalence.

**Fig 1:**
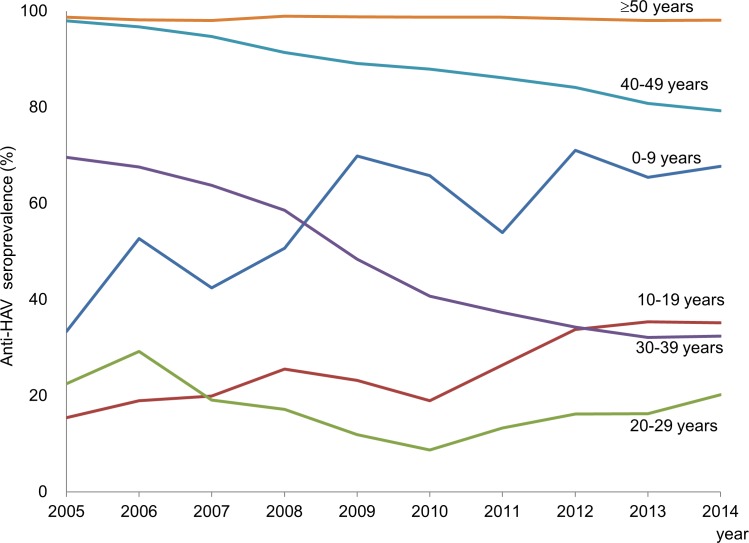
Changes of seroprevalence of anti-HAV according to age group from 2005 to 2014.

**Fig 2:**
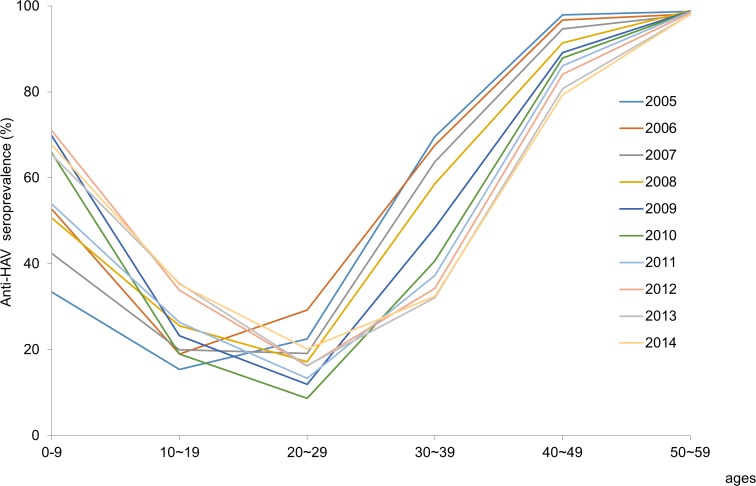
Age-specific seroprevalence of anti-HAV adjusted by area and gender from 2005 to 2014.

Persons aged 0 to 9 years showed a remarkable increase of anti-HAV prevalence over the 10-year period. Anti-HAV prevalence in teens gradually increased from 15.4% to 35.2% over the 10 years. The increase of anti-HAV prevalence in persons below 20 years of age may have resulted from vaccination. However, seroprevalence in persons aged 20 to 29 years decreased from 22.5% in 2005 to 8.7% in 2010 rebounding to 20.2% in 2014. (Fig 1) In contrast, seroprevalence abruptly decreased in persons aged 30 to 39 years (from 69.6% to 32.4%). In those aged 40 to 49 years, the seroprevalence gradually decreased from 97.9% to 79.3% over the 10 years. The dynamic changes in persons aged 20 to 49 years were attributable to the cohort effect. The seroprevalence in persons older than 50 years showed more than 98% which remained unchanged during the study period. Therefore, the population most susceptible to HAV infection at present consists of persons aged 20 to 39 years, the birth cohort which were born from the mid-1970s to mid-1990s. (Fig 2)

Seroprevalence of anti-HAV by age, area and year was shown in S1 Table. It was significantly different among 5 or 6 areas for both the first and latter five years (p<0.05). Age- and gender-adjusted anti-HAV seroprevalence was much higher in Jeolla & Jeju and Gangwon where rural areas are predominant. Unadjusted area-specific seroprevalence of persons aged 30~49 years was highest in Jeolla & Jeju and lowest in Seoul. The anti-HAV seroprevalence in persons aged 30 to 39 years dropped below 50% 4 year earlier in Seoul (2008) than in rural areas (2012). In persons aged 40 to 49 years it dropped dropped below 90% 4 year earlier in Seoul (2009) than in rural areas (2013).

Seroprevalence by gender was also analyzed for the latter 5 years, because limited sample size during the first 5 years did not permit gender-adjusted analysis. Area- and age-adjusted seroprevalence was higher in females than in males across the five years. (Table 3), which may be attributable to the higher proportion of males in persons below 20 years who have low anti-HAV positivity and the higher proportion of females in persons over 60 years who have near 100% anti-HAV positivity. The seroprevalence in persons aged 20–29 years was slightly higher in females than in males. In contrast, the seroprevalence in persons in their thirties was slightly higher in male than in female.

**Table 3:** Seroprevalence of anti-HAV by sex.

	2010		2011		2012		2013		2014	
Age	M	F	M	F	M	F	M	F	M	F
0~9	61.8	65.4	45.2	56.8*	65.2	69.7	62	66.5	67.5	65.4
10~19	18.1	20	25.7	26.5	31.1	33.1	35.3	35.1	37.1	31.9*
20~29	7.9	9.6*	12.3	13.9*	15.3	16.3	14.8	17.5*	16.5	19.9*
30~39	42.6	38*	38.6	32.9*	35.6	30.4*	33.4	29.4*	31	30.5
40~49	87.2	88.4*	85.4	86.3	84	84.4	81.3	81.6	79.9	79.9
50~59	98.7	98.7	98.6	98.7	98.2	98.4	97.9	98.2	97.9	98.2
60~	98.8	99.4	97.7	98.9*	99	99.5	99.5	99.7	99.3	99.7*
Total**	59.3	62.7	58.3	62.6	61.5	64.2	60.5	62.7	61.0	63.2

*Positivity of anti-HAV was significantly different between males and females (p < .05 by the chi-square test)

**Seroprevalence of anti-HAV was adjusted by age and area.

## Discussion

This study investigated the nationwide prevalence of anti-HAV adjusted by age, gender and area from 2005 to 2014, a period including the nationwide outbreak of hepatitis A that peaked in 2009, by analyzing the results from a total of 424,245 anti-HAV sera from one central laboratory to which serologic tests for HAV were referred by medical institutions around the nation. The overall nationwide seroprevalence rates of anti-HAV gradually decreased during the last 10 years, from 65.6% to 62.2%, and age-specific seroprevalence showed dynamic changes. Seroprevalence continuously decreased in person aged 30 to 49 years due to the cohort effect; the segment of the population with natural HAV immunity was progressively replaced by birth cohorts with fewer early childhood HAV exposures and with no natural immunity. In contrast, it increased in persons aged 0 to 19 years during the 10-year period probably owing to vaccination. In persons aged 20 to 29 years, it reached a trough in 2010 and thereafter started to increase. This may have been influenced by both the cohort effect and vaccination. Therefore, in persons aged 20 to 39 years, the birth cohort born between the mid-1970s and mid-1990s and whose anti-HAV seroprevalence is still less than 50%, the risk of an HAV outbreak with clinically severe manifestations has increased.

Until the early 1980s, HAV infection mainly occurred asymptomatically in early childhood with subsequent lifelong immunity in almost all Korean adults.[7] As socioeconomic status and sanitary conditions improved, anti-HAV positivity decreased to less than 10% in children and 20–30% in young adolescents in the mid-2000s.[8, 9] The seroepidemiologic shift resulted in a great national outbreak of hepatitis A that peaked in 2009.[10, 11] The age and area-adjusted overall seroprevalence rates of anti-HAV from 2010 to 2014 was lower than those from 2005 to 2009. This resulted from the marked decrease of seroprevalence in persons aged 30 to 49 years throughout the 10-year period.

The positivity of anti-HAV in persons aged below 10 years tended to increase over the 10-year period of this study. It dropped to as low as 2% in the late 1990s,[12] but increased to 33.4% in 2005 and plateaued at 62.8~69.9% in the late 2000s.[6, 9] Considering the amount of vaccine sales and the active campaign of pediatric societies advocating HAV vaccination during the last 10 years, [8, 13] the increased positivity of IgG anti-HAV in children from 33.4% in 2005 to 67.7% in 2014 can be explained by increased HAV vaccination. Likewise, the positivity of IgG anti-HAV in persons aged 10 to 19 years increased from 2005 (15.4%) to 2014 (35.2%), though to a lesser extent than those of children aged under 10 years old. As of May 2015, the KCDC has included HAV to the national vaccination program for children. Thus, seroprevalence of the child population is expected to reach nearly 100% in the next 10 years. Meanwhile, as of 2012 the Korean armed forces have been providing HAV vaccination for about 30,000 new recruits every year especially for those on kitchen duty in the army.[14] Therefore, the incidence of hepatitis A will decrease further particularly in adolescents and young people, and the seroepidemiology of HAV will keep changing.

Young adults aged 20 to 29 years showed the lowest positivity of IgG anti-HAV, and this figure has not so changed remarkably during the last 10 years. On the contrary, positivity of anti-HAV in persons aged 30 to 39 continuously decreased from 69.6% in 2005 to 32.4% in 2014, and positivity of anti-HAV in persons aged 40 to 49 years decreased more slowly from 97.9% to 79.3% throughout the 10- year period, showing an epidemiological shift of HAV, which was observed in many developed countries.[15, 16]

Age- and gender-adjusted prevalence of anti-HAV was higher in Jeolla/Jeju and Gangwon which include more rural areas than in Seoul, the capital of Korea. This geographic difference could also result from a higher proportion of elderly subjects in these areas. However, crude area-specific prevalence of persons in their 40s was also much higher in Jeolla/Jeju than in Seoul (87.5% vs 75.0% in 2014). The anti-HAV seroprevalence in persons in their thirties dropped below 50% 4 years earlier in Seoul (2008) than in rural areas (2012). In persons aged 40 to 49 years it dropped below 90% 4 years earlier in Seoul (2009) than in rural areas (2013). The speed of urbanization may have caused the time lag between urban and rural areas in regards to the replacement of an immune cohort by a non-immune cohort unexposed to HAV during their early childhood. Seroepidemiologic differences between urban and rural areas as well as among income levels were also observed in previous studies.[17, 18]

The age- and area-adjusted seroprevalence was higher in females than in males. We guessed this difference may have resulted from the older age distribution in female. However, age-specific seroprevalence also showed gender difference. The seroprevalence of anti-HAV in females aged 20 to 29 years was statistically lightly higher than in male. The reason for higher seroprevalence in females in their twenties was not clear, but active prenatal screening for HAV immununity and subsequent vaccination may have caused this difference. In addition, some women of this age group may be involved in childcare and thus exposed to young children and obtain natural immunity. In contrast, the seroprevalence in males in their thirties was slightly higher than in females. A higher incidence of hepatitis A in young males during the epidemic in Korea[10] may have resulted in the higher seroprevalence of males in their thirties in this study.

The strengths of our study were the large number of study subjects and the fact that it was conducted nationwide for a period of 10 years. However, our study has some limitations. First, this study analyzed the samples referred by medical institutions, which cannot exactly represent the general population of South Korea. Actually, this result may be considered as a significant sentinel tool for the monitoring of the annual changes in anti-HAV in this population rather than representing the true prevalence of anti-HAV. Because there is no available way to assess the true seroprevalence of HAV in South Korea, this study may be a representative surrogate of it, considering the large number and nationwide distribution of the study population. In addition, some might request IgG anti-HAV after vaccination for reassurance, provoking possible overestimation of prevalence in young children, though retesting after vaccination in immunocompetent hosts is not recommended for HAV vaccination. Second, more than 30% of the serologic results were excluded due to lack of demographic information, but this probably had little influence on the results, considering that the crude anti-HAV positivity in the excluded individuals was nearly the same as that of the included individuals.

After the epidemic of hepatitis A peaked in 2009 in South Korea (15,231 cases from the sentinel surveillance system), occurrence of hepatitis A markedly decreased to 5,521 cases in 2011 and 1,307 cases in 2014. However, persons with HAV infection are becoming older. According to the KCDC’s infectious disease surveillance yearbook, proportions of hepatitis A cases in persons in their twenties (from 37.1% to 25.1%) and thirties (from 43.4% to 41.7%) decreased, whereas hepatitis A in persons aged 40~49 years increased from 10.5% in 2009 to 22.0% in 2014. Therefore, persons aged 20~39 years who were born between the mid-1970s and mid-1990s are still the most vulnerable to hepatitis A. As this birth cohorts are older, HAV infection in persons aged over 40 years will continue to increase. The severity of hepatitis A increased in persons with chronic liver disease or old age.[11, 19, 20] Taking this changing seroprevalence into account, more severe clinical presentations in older patients and subsequent rise in morbidity and mortality can be expected in the near future. The mortality rate of acute hepatitis A was reported to be 0.47%, and acute renal failure developed in 2.7% of hepatitis A cases during the outbreak in South Korea.[11]

In conclusion, from 2005 to 2014, the seroprevalence of hepatitis A has been changing dynamically in Koreans aged below 50 years, and persons aged 20 to 39 years who were born between the mid-1970s and mid-1990s are still the most vulnerable to HAV infection. Tests for HAV immunity and vaccination should be considered for this birth cohort, especially those with high risks for HAV infection or chronic liver disease. Although the Korean government initiated a national hepatitis A immunization program for infants in 2015, the immunity of young adults is still decreasing, and an outbreak of HAV is possible in the near future. Therefore, continuous monitoring and optimal preventive measures to prevent such an outbreak should be considered.

## Supporting Information

S1 Table Anti-HAV seroprevalence by age, area and year*. HAV, hepatitis A virus.
*Positivity of anti-HAV was significantly different among five areas from 2005 to 2009 across ages and also significantly different among six areas from 2010 to 2014 across ages. (p < .05 by Chi-square test)
**Overall seroprevalence of anti-HAV was adjusted by age from 2005 to 2009 and by age and sex from 2010 to 2014
†Gyeonggi +Incheon and Gangwon were grouped together from 2005 to 2009 considering a small number of tests and geological proximity.
(DOCX)
