# Supplementary material for: Nationwide Seropositivity of Hepatitis A in Republic of Korea from 2005 to 2014, before and after the Outbreak Peak in 2009
Source: PLoS One. 2017 Jan 18;12(1):e0170432. doi: 10.1371/journal.pone.0170432 (PMC5242508; doi:10.1371/journal.pone.0170432)
Supplement: S1 Table — HAV, hepatitis A virus. *Positivity of anti-HAV was significantly different among five areas from 2005 to 2009 across ages and also significantly different among six areas from 2010 to 2014 across ages. (p < .05 by Chi-square test) **Overall seroprevalence of anti-HAV was adjusted by age from 2005 to 2009 and by age and sex from 2010 to 2014 †Gyeonggi +Incheon and Gangwon were grouped together from 2005 to 2009 considering a small number of tests and geological proximity. (DOCX) [file pone.0170432.s001.docx]

S1 Table. Anti-HAV seroprevalence by age, area and year*

| Area | Age | Year | | | | | | | | | |
| --- | --- | --- | --- | --- | --- | --- | --- | --- | --- | --- | --- |
|  |  | 2005 | 2006 | 2007 | 2008 | 2009 | 2010 | 2011 | 2012 | 2013 | 2014 |
| Seoul | 0~9 | 16.7 | 58.3 | 51.1 | 38.9 | 67.3 | 70.2 | 57.9 | 66.7 | 62.4 | 66.1 |
|  | 10~19 | 21.4 | 21.1 | 20.9 | 25.9 | 25.4 | 28.5 | 33.3 | 41.3 | 44.6 | 41.0 |
|  | 20~29 | 17.6 | 27.4 | 16.7 | 14.2 | 12.9 | 10.8 | 15.8 | 21.9 | 21.6 | 24.4 |
|  | 30~39 | 69.1 | 59.7 | 55.1 | 48.7 | 39.0 | 35.2 | 34.6 | 32.6 | 31.4 | 33.1 |
|  | 40~49 | 97.4 | 94.3 | 93.2 | 91.5 | 80.0 | 83.4 | 80.7 | 79.7 | 76.3 | 75.0 |
|  | 50~59 | 99.0 | 99.4 | 99.2 | 98.7 | 96.5 | 97.7 | 98.0 | 98.5 | 97.1 | 97.1 |
|  | 60+ |  |  |  |  |  | 98.6 | 98.1 | 99.0 | 99.3 | 99.3 |
|  | Overall** | 63.6 | 66.6 | 63.2 | 60.8 | 59.2 | 59.3 | 58.9 | 61.5 | 60.6 | 61.2 |
| Gyeonggi & Incheon | 0~9 | 30.0 | 52.6 | 40.9 | 47.4 | 69.9 | 71.3 | 57.7 | 84.1 | 76.1 | 80.0 |
|  | 10~19 | 22.9 | 16.1 | 13.2 | 18.3 | 17.8 | 19.2 | 30.6 | 39.0 | 36.4 | 42.0 |
|  | 20~29 | 22.9 | 16.1 | 13.2 | 18.3 | 17.8 | 8.9 | 14.4 | 17.0 | 18.7 | 21.3 |
|  | 30~39 | 18.9 | 30.2 | 14.5 | 15.3 | 10.4 | 36.1 | 33.2 | 33.6 | 33.9 | 32.8 |
|  | 40~49 | 99.0 | 94.9 | 95.0 | 92.8 | 91.5 | 88.2 | 86.2 | 84.7 | 80.1 | 77.3 |
|  | 50~59 | 99.0 | 99.0 | 99.3 | 99.5 | 99.5 | 98.8 | 98.9 | 98.5 | 98.3 | 98.3 |
|  | 60+ |  |  |  |  |  | 99.5 | 95.6 | 100 | 99.6 | 99.7 |
|  | Overall** | 64.0 | 67.4 | 62.0 | 62.8 | 62.5 | 59.6 | 59.3 | 63.6 | 60.9 | 61.9 |
| Gangwon† | 0~9 |  |  |  |  |  | 64.4 | 38.5 | 52.5 | 82.4 | 67.1 |
|  | 10~19 |  |  |  |  |  | 17.2 | 14.8 | 23.0 | 49.1 | 47.4 |
|  | 20~29 |  |  |  |  |  | 7.9 | 18.4 | 16.3 | 13.1 | 17.0 |
|  | 30~39 |  |  |  |  |  | 43.0 | 44.7 | 38.6 | 33.1 | 33.7 |
|  | 40~49 |  |  |  |  |  | 89.4 | 87.7 | 84.5 | 85.3 | 78.4 |
|  | 50~59 |  |  |  |  |  | 98.8 | 100 | 99.7 | 98.6 | 99.2 |
|  | 60+ |  |  |  |  |  | 100 | 100 | 98.3 | 100 | 98.9 |
|  | Overall** |  |  |  |  |  | 64.5 | 63.2 | 63.7 | 69.0 | 66.8 |
| Chungcheong | 0~9 |  |  |  |  |  | 59.0 | 56.4 | 61.8 | 50.7 | 52.0 |
|  | 10~19 | 9.1 | 35.3 | 21.1 | 50.0 | 21.4 | 14.3 | 22.1 | 29.9 | 32.1 | 31.2 |
|  | 20~29 | 11.1 | 43.8 | 28.6 | 9.7 | 12.2 | 8.3 | 11.6 | 13.4 | 15.1 | 19.6 |
|  | 30~39 | 40.0 | 62.5 | 71.1 | 72.4 | 47.8 | 44.1 | 40.7 | 40.0 | 37.5 | 38.6 |
|  | 40~49 | 99.0 | 99.0 | 99.0 | 83.6 | 88.9 | 86.8 | 84.7 | 81.7 | 80.4 | 80.1 |
|  | 50~59 | 99.0 | 96.0 | 97.0 | 99.0 | 98.9 | 98.4 | 98.6 | 97.8 | 97.4 | 97.5 |
|  | 60+ |  |  |  |  |  | 98.7 | 97.9 | 100 | 99.7 | 99.4 |
|  | Overall** | 59.6 | 70.9 | 66.2 | 69.4 | 62.5 | 59.9 | 60.2 | 62.0 | 60.5 | 62.5 |
| Gyeongsang | 0~9 | 43.3 | 46.9 | 44.8 | 54.9 | 68.8 | 64.9 | 48.8 | 68.9 | 67.4 | 70.3 |
|  | 10~19 | 2.3 | 12.3 | 23.1 | 20.8 | 26.2 | 16.1 | 22.2 | 26.1 | 30.2 | 31.2 |
|  | 20~29 | 25.8 | 23.9 | 15.0 | 25.8 | 13.3 | 8.7 | 10.6 | 12.5 | 14.1 | 13.6 |
|  | 30~39 | 84.4 | 63.5 | 61.9 | 54.6 | 47.1 | 40.9 | 31.3 | 26.0 | 24.1 | 23.7 |
|  | 40~49 | 96.2 | 99.0 | 94.9 | 92.0 | 89.7 | 88.3 | 87.6 | 84.7 | 81.3 | 79.3 |
|  | 50~59 | 98.1 | 99.0 | 98.4 | 98.5 | 99.7 | 99.0 | 98.7 | 98.1 | 98.1 | 98.1 |
|  | 60+ |  |  |  |  |  | 99.1 | 99.0 | 98.6 | 99.4 | 99.7 |
|  | Overall** | 68.3 | 67.3 | 66.4 | 67.0 | 66.0 | 62.3 | 60.7 | 61.7 | 61.6 | 61.2 |
| Jeolla & Jeju | 0~9 | 37.8 | 59.1 | 45.0 | 59.4 | 76.0 | 51.8 | 50.0 | 58.2 | 55.7 | 62.2 |
|  | 10~19 | 20.0 | 23.5 | 29.4 | 33.3 | 30.1 | 15.2 | 22.2 | 32.1 | 30.5 | 25.6 |
|  | 20~29 | 50.0 | 28.6 | 40.0 | 16.8 | 10.5 | 6.8 | 14.8 | 16.4 | 10.9 | 20.4 |
|  | 30~39 | 81.8 | 88.2 | 88.9 | 71.7 | 69.2 | 56.5 | 52.1 | 45.6 | 38.3 | 39.9 |
|  | 40~49 | 99.0 | 99.0 | 91.7 | 92.4 | 96.8 | 94.0 | 92.1 | 90.8 | 88.5 | 87.5 |
|  | 50~59 | 99.0 | 94.4 | 92.9 | 99.0 | 98.8 | 99.8 | 99.0 | 98.8 | 98.8 | 98.4 |
|  | 60+ |  |  |  |  |  | 99.4 | 99.1 | 99.5 | 100 | 99.4 |
|  | Overall** | 73.3 | 72.7 | 71.9 | 70.9 | 71.6 | 64.6 | 65.1 | 66.5 | 64.2 | 65.3 |

HAV, hepatitis A virus.

*Positivity of anti-HAV was significantly different among five areas from 2005 to 2009 across ages and also significantly different among six areas from 2010 to 2014 across ages. (p<.05 by Chi-square test)

**Overall seroprevalence of anti-HAV was adjusted by age from 2005 to 2009 and by age and sex from 2010 to 2014

†Gyeonggi +Incheon and Gangwon were grouped together from 2005 to 2009 considering a small number of cases and geological proximity.
